# Office Visitors

**Published:** 1907-11

**Authors:** 


### OFFICE VISITORS.

He was of the small farmer variety, who had left the range of our practice and after many years was returning to his native heath to spend the remainder of his days. As he passed through the city he was so pleased to "meet up" with some of his old neighbors, that they had to celebrate the occasion with sundry inbibations of John Barleycorn. He was told that we had an office in one of the prominent office-buildings and he proceeded to "hunt us up." He found us and his greeting was, "Hello, Doc, does yer know me? I reckon not and I wouldn't er knowd you if I hadn't been tole you was in here, an I seed yer name on ther do glass—weve bofe got grizzly. How air yer, and whats ther good word. I ain't seed yer befo in nigh onter twenty year. And so you've lef the ole town an cum over to ther city, eh? Well I'm glad I've found yer out whilst I was er passin thro. Yes, I'm er goin back ter ther ole neighborhood. Yes, yer see hit was sickly out thar, but the lan was rich and we made fine craps, but we hed er lot er chills an fever. We managed ter git erlong widout er death in the family and raised er haf dozen chillun, but we've altergether swallered er barrel of quinine. Well, ther chillun is all grode up an married off an ther whole passel have moved er long way off an me an the ole woman got lonesum like, an we got ter thinking of ther old place an ther ole kinfolks, so we just sold out an am on our way back ter ole Carlina fer good an ter stay ther till we die an be buried thar. But befo I gits under ther groun, I wants yer ter make over mer set of teeth. Yer made em forty years ago. Yer aint that ole you say? Well how yer cum by that ar gray har on yer hed. Well hits been all of thirty year an no joke. Hits been er good set, but hit orto er bin, fer yer charged me twenty dollars fer em. Now wat yer goin ter charge me for makin em over? They needs refixin es mer gums is swunk sum een that time. Now be lite. This ere set has brung you een lots er wuk, fer befo I lef ther state, an moved ter Gorgy I bragged on em powerful, an sent yer er menny er one. Sum time ergo I had er feller, er travelin tooth dentis ter pull out ther ole woman's teeth an mek her er set, an he only charged four dollers, min yer, er bran new full set er top ones. Now these er mine is jes er make over job, wid ther same teeth. I wouldn't let no body tech em, fer I lowed sum day ter git back ter yer, fer yer ter do ther work, an yere I is. Now set yer price low as possible an tek mer mesure." A satisfactory price was agreed on for old times sake and we proposed to take impressions when from his coat pocket he brought forth a flat pint bottle of the aforementioned J. B. Corn and reckoned he'd better take "er swaller to keep fum gaggin wen thet mortar gits een mer mouth." Of course, the mouth of the bottle was offered to us invitingly. Not to seem unfriendly we went thro' the motion by blowing bubbles into the bottle. The "mesure was tuck" and we sat down to talk of the old times and the old people. Who he reckoned he'd know and who would know him. We enumerated to him who had died and who were now living and were much interested in the theme when we were startled by a female figure at our office door, who abruptly came on the scene, exclaiming: "Why howdy Doctor, I'm so glad ter see yer, but I've been er huntin yer, ole man, fer ther las two hours. We mus go, haint got but ten minutes ter git to ther cyar. Shake hands with the Doctor." We shook them.

